# Methylome analysis and whole-exome sequencing reveal that brain tumors associated with encephalocraniocutaneous lipomatosis are midline pilocytic astrocytomas

**DOI:** 10.1007/s00401-018-1898-8

**Published:** 2018-08-24

**Authors:** Elvis Terci Valera, Melissa K. McConechy, Tenzin Gayden, Barbara Rivera, David T. W. Jones, Andrea Wittmann, HyeRim Han, Eric Bareke, Hamid Nikbakht, Leonie Mikael, Rosane Gomes Queiroz, Veridiana Kiill Suazo, Ji Hoon Phi, Seung-Ki Kim, Sung-Hye Park, Raita Fukaya, Mi-Sun Yum, Tae-Sung Ko, Ricardo Santos de Oliveira, Helio Rubens Machado, María Sol Brassesco, Antonio Carlos do Santos, Gustavo Novelino Simão, Leandra Náira Zambelli Ramalho, Luciano Neder, Carlos Alberto Scrideli, Luiz Gonzaga Tone, Jacek Majewski, Nada Jabado

**Affiliations:** 1 Department of Pediatrics, Ribeirão Preto Medical School, University of São Paulo, HC Criança-Av. Bandeirantes, 3900, Ribeirão Preto, SP CEP 14048-900 Brazil; 2 Department of Human Genetics, McGill University, Montreal, QC Canada; 3 Department of Pediatrics, The Research Institute of the McGill University Health Center, McGill University, Montreal, QC H4A 3J1 Canada; 4 Pediatric Glioma Research Group, Hopp Children’s Cancer Center, NCT Heidelberg (KiTZ) and German Cancer Research Center (DKFZ), Heidelberg, Germany; 5 McGill University and Genome Quebec Innovation Center, Montreal, QC Canada; 6 Division of Pediatric Neurosurgery, Seoul National University Children’s Hospital, Seoul, Republic of Korea; 7 Department of Pathology, Seoul National University Children’s Hospital, Seoul, Republic of Korea; 8 Department of Neurosurgery, Shizuoka City Shimizu Hospital, Shizuoka, Japan; 9 Department of Neurosurgery, Fuji Hospital, Aichi, Japan; 10 Division of Pediatric Neurology, Department of Pediatrics, Asan Medical Center Children’s Hospital, University of Ulsan College of Medicine, Seoul, Republic of Korea; 11 Division of Pediatric Neurosurgery, Department of Surgery and Anatomy, University Hospital, Ribeirão Preto Medical School, University of São Paulo, Ribeirão Preto, SP Brazil; 12 Department of Biology, Faculty of Philosophy, Sciences and Letters at Ribeirão Preto, University of São Paulo, Ribeirão Preto, SP Brazil; 13 Department of Image Science and Medical Physics Center, Internal Medicine, University of São Paulo, Ribeirão Preto, SP Brazil; 14 Department of Pathology, Ribeirão Preto Medical School, University of São Paulo, Ribeirão Preto, Brazil

**Keywords:** Encephalocraniocutaneous lipomatosis, *FGFR1*, RASopathies, genetics, brain tumors, Children

## Introduction

Encephalocraniocutaneous lipomatosis (ECCL;[MIM:613001]) is a rare sporadic RASopathy due to one of two mutually exclusive fibroblast growth factor receptor 1 (FGFR1) mutations p.N546K or p.K656E. These activating hotspot mutations are identified in affected tissues, but not in the peripheral blood of ECCL patients, and are likely the result of post-zygotic constitutional mosaicism promoting locally constitutive activation of the RAS-MAPK pathway [1]. The same FGFR1 mutations occur in subgroups of sporadic low-grade gliomas (LGG) [7, 10, 12] indicating probable intersection between ECCL and tumorigenesis, possibility further substantiated by reports of brain tumors in nine ECCL cases with wide-ranging histopathological subtypes [1–3, 5, 6, 8, 9, 13].

**Fig. 1:**
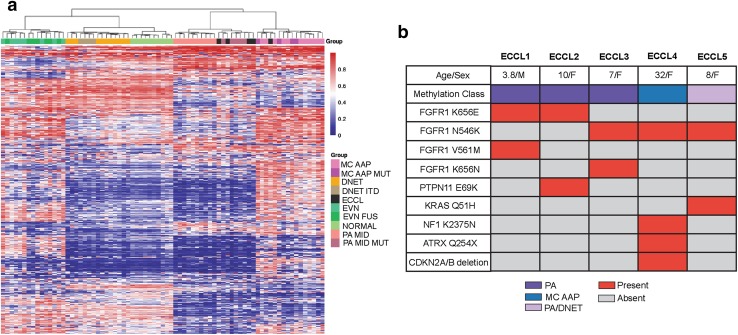
DNA methylation classification and mutations identified in five ECCL-associated brain tumors. **a** Hierarchical clustering of methylation data from five ECCL tumors (black) with 75 reference cases of nine established glioma methylation classes indicated by different colors. Reference classes: MC-AAP methylation class anaplastic astrocytoma with piloid features; MC-AAP MUT with *FGFR1* mutation; DNET dysembryoplastic neuroepithelial tumor; DNET ITD internal duplication of *FGFR1*; EVN extraventricular neurocytoma; EVN FUS with *FGFR1:TACC1* fusion; NORMAL normal brain; PA MID midline pilocytic astrocytoma; PA MID MUT with *FGFR1* mutation. **b** Summary of ECCL patient clinical and molecular characteristics. Red boxes indicate presence and gray boxes absence of a given genetic alteration

To evaluate the pathological and genetic landscape of these brain tumors in ECCL, we acquired five of these cases (Suppl. Table 1 Online Resource 1 and 4), representative H&E and MRI for each provided in Suppl. Fig. 1 (Online Resource 3). Four were originally reported as LGG, either pilocytic astrocytomas (PA) (ECCL1, ECCL2) [3, 13], papillary glioneural tumor (PGNT) (ECCL3) [9], or dysembryoplastic neuroepithelial tumor (DNET) (ECCL5) [6], while ECCL4 was reported as a glioblastoma [5]. Blinded histopathological review resulted in re-classification of the PGNT/ECCL3 as a pilomyxoid astrocytoma (PMA), and the DNET/ECCL5 as PA. DNA methylation analysis [4] using hierarchical clustering and t-SNE analysis with 75 reference cases representing nine tumor subclasses [11] revealed that three out of five tumors are midline PAs, and subcluster with *FGFR1*-mutated midline PAs (Fig. 1a; Suppl. Fig. 2 Online Resource 3): ECCL1 and ECCL2 showed high classifier scores for PA (0.98 and 1.00, respectively). ECCL3 had a low score (0.09) likely due to normal tissue, but still reliably clustered with PAs. ECCL4 clustered with the rare subgroup of recently described methylation class anaplastic astrocytoma with piloid features (MC-AAP) [11], a classification further substantiated by the *CDKN2A/B* deletion identified in this sample (Suppl. Fig. 3 Online Resource 3). ECCL5 received the highest methylation classifier score for PA (0.43). Hierarchical clustering further suggested an *FGFR1*-mutated midline PA, while on t-SNE analysis, this tumor resembled DNETs (Suppl. Fig. 2 Online Resource 3), mirroring the histological dilemma between DNET and PA for this tumor, two entities of the spectrum of *FGFR1*-mutant brain tumors.

Whole-exome sequencing on these five tumors and matched peripheral blood available from three patients (ECCL1, 2, 4) identified *FGFR1* K656E (ECCL1, ECCL2) and *FGFR1* N546K (ECCL3, ECCL4, ECCL5) (Suppl. Fig. 4 Online Resource 3, Suppl. Table 2 Online Resource 2). All five tumors showed additional concurrent alterations in FGFR1/RAS/MAPK pathway genes, including *NF1*, *KRAS, PTPN11,* and *FGFR1* mutations (Fig. 1b). Two cases harbored a second mutation in *FGFR1*: ECCL3 had confirmed *in cis FGFR1* N546K/K656N mutations (Suppl. Fig. 5 Online Resource 3); ECCL1 had concurrent somatic *FGFR1* K656E/V561M mutations possibly also *in cis* based on a previous report of similar *in cis FGFR1* combination in an ECCL PA [1], even if we could not confirm this due to unavailability of material. ECCL2 had *PTPN11* E69K and ECCL5 *KRAS* Q61H mutations, both previously identified in sporadic PAs [11, 14]. Also, co-occurence of FGFR1/PTPN11 mutations has been described in a small subset of PAs [7]. In ECCL4, we identified two additional somatic *NF1* K2375N and *ATRX* Q254X mutations (Fig. 1b, Suppl. Table 2 Online Resource 2), a pattern which, in addition to *CDKN2B/A* deletion, the high-grade histological features and older age of ECCL4 is concordant with what has been described in MC-AAPs [11].

Finally, somatic mosaicism and non-hereditary nature of *FGFR1* mutations in ECCL patients and their parents were confirmed in two cases using targeted sequencing. In ECCL1, *FGFR1* mutations were absent in blood DNA in the patient and mother (Suppl. Fig. 6 Online Resource 3, Suppl. Table 2 Online Resource 2). In ECCL2, co-occurrence of *FGFR1* and *PTPN11,* mutations were exclusive to the brain tumor while the skin lipoma had only the *FGFR1* mutation, suggesting the need for a “second hit” in the MAPK pathway in the brain (Suppl. Fig. 6 Online Resource 3).

In summary, integrating histology and molecular data on the largest cohort of ECCL-associated brain tumors assembled to date shows that these are midline PAs. A degree of glioneuronal differentiation may lead to a diagnosis of DNET, while ECCL4 originally diagnosed as glioblastoma would have been diagnosed as MC-AAP based on recent findings. The initial *FGFR1* mutation requires additional somatic alterations in the FGFR1/RAS/MAPK pathway to drive tumorigenesis towards development of distinct subgroups of PAs in ECCL. Thus, even if additional molecular follow-up studies are needed to confirm these observations, pathogenesis of ECCL-associated PA is possibly distinct from that of sporadic PAs where typically one hit is needed [8]. Moreover, the use of novel therapies targeting FGFR1 may prove less effective as some of these second hits are downstream of the receptor. In conclusion, our data reinforce the acquired genetic trait and mosaic nature of ECCL and further emphasize the need for in-depth molecular analysis to refine and ensure accuracy of pathological diagnosis and clinical decision-making approaches for affected patients.

## Electronic supplementary material

Below is the link to the electronic supplementary material.

Supplementary material 1 (XLSX 16 kb)

Supplementary material 2 (XLSX 110 kb)

Supplementary material 3 (PDF 6230 kb)

Supplementary material 4 (PDF 179 kb)
