# Supplementary material for: Methylome analysis and whole-exome sequencing reveal that brain tumors associated with encephalocraniocutaneous lipomatosis are midline pilocytic astrocytomas
Source: Acta Neuropathol. 2018 Aug 24;136(4):657–60. doi: 10.1007/s00401-018-1898-8 (PMC6132939; doi:10.1007/s00401-018-1898-8)
Supplement: Supplementary file 3 — Supplementary material 3 (PDF 6230 kb) [file 401_2018_1898_MOESM3_ESM.pdf]

Methylome analysis and whole exome sequencing reveal that brain tumors associated with encephalocraniocutaneous lipomatosis are midline pilocytic astrocytomas

Elvis Terci Valera[1,2*], Melissa K. McConechy[2*], Tenzin Gayden[3*], Barbara Rivera[2], David T. W. Jones[4], Andrea Wittmann[4], HyeRim Han[2], Eric Bareke[5], Hamid Nikbakht[5], Leonie Mikael[3], Rosane Gomes Queiroz[1], Veridiana Kiill Suazo[1], Ji Hoon Phi[6], Seung-Ki Kim[6], Sung-Hye Park[7], Raita Fukaya[8,9], Mi-Sun Yum[10], Tae-Sung Ko[10], Ricardo Santos de Oliveira[11], Helio Rubens Machado[11], María Sol Brassesco[12], Antonio Carlos do Santos[13], Gustavo Novelino Simão[13], Leandra Náira Zambelli Ramalho[14], Luciano Neder[14], Carlos Alberto Scrideli[1], Luiz Gonzaga Tone[#1], Jacek Majewski[#2,5], Nada Jabado[#2,3]

[1]Department of Pediatrics, Ribeirão Preto Medical School, University of São Paulo, Ribeirão Preto, São Paulo, Brazil.

[2]Department of Human Genetics, McGill University, Montreal, Quebec, Canada

[3]Department of Pediatrics, The Research Institute of the McGill University Health Center, Montreal, Quebec, Canada.

[4]Pediatric Glioma Research Group, Hopp Children's Cancer Center at the NCT Heidelberg (KiTZ) and German Cancer Research Center (DKFZ), Heidelberg, 69120, Germany.

[5]McGill University and Genome Quebec Innovation Center, Montreal, Quebec, Canada.

[6]Division of Pediatric Neurosurgery, Seoul National University Children's Hospital, Seoul, Republic of Korea.

[7]Department of Pathology, Seoul National University Children's Hospital, Seoul, Republic of Korea.

[8]Department of Neurosurgery, Shizuoka City Shimizu Hospital, Shizuoka, Japan

[9]Department of Neurosurgery, Fuji Hospital, Aichi, Japan

[10]Division of Pediatric Neurology, Department of Pediatrics, Asan Medical Center Children's Hospital, University of Ulsan College of Medicine, Seoul, Republic of Korea

[11]Division of Pediatric Neurosurgery, Department of Surgery and Anatomy, University Hospital, Ribeirão Preto Medical School, University of São Paulo, Ribeirão Preto, São Paulo, Brazil

[12]Department of Biology, Faculty of Philosophy, Sciences and Letters at Ribeirão Preto, University of São Paulo, Ribeirão Preto, São Paulo, Brazil

[13]Department of Image Science and Medical Physics Center, Internal Medicine, University of São Paulo, Ribeirão Preto, São Paulo, Brazil.

[14]Department of Pathology, Ribeirão Preto Medical School, University of São Paulo, Ribeirão Preto, Brazil.

[*] Equal contribution

[#] co-senior authors

To whom correspondence should be sent:

Elvis Terci Valera, MD, PhD
Department of Pediatrics
Ribeirão Preto Medical School, University of São Paulo, São Paulo, Brazil.

HC Criança - Av. Bandeirantes, 3900, Ribeirão Preto, SP CEP 14048-900 Brazil.

valeraet@gmail.com

Nada Jabado, MD, PhD
Department of Pediatrics
The Research Institute of McGill University Health Center
McGill University
Montreal, QC, H4A 3J1, Canada
nada.jabado@mcgill.ca

Keywords: Encephalocraniocutaneous Lipomatosis; *FGFR1*; RASopathies, genetics, brain tumors; children

This pdf File contains Supplementary Figures 1-6

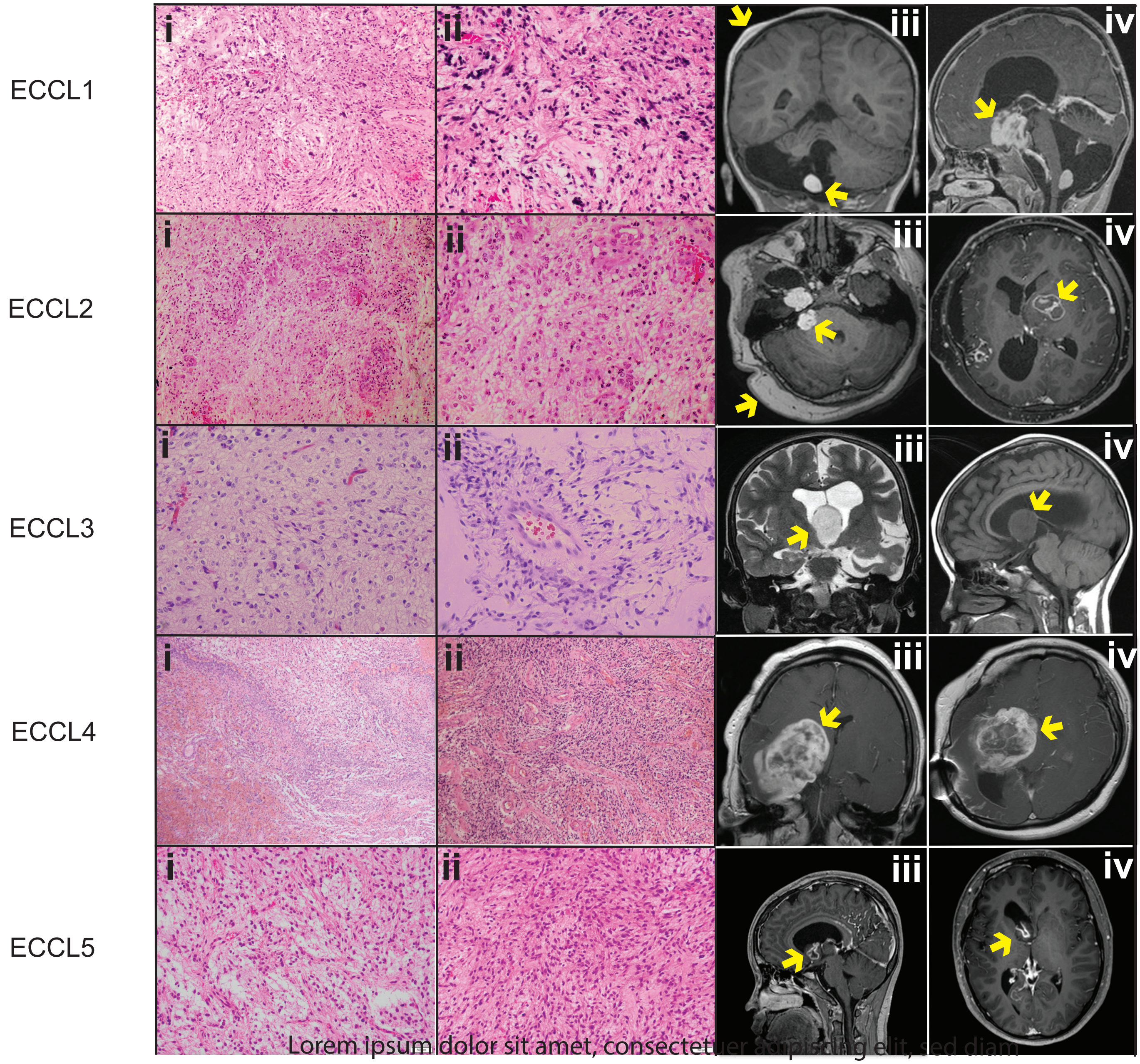

Suppl. Fig. 1 Five panels in parallel for each ECCL case showing representative H&E stained sections (i, ii), and T1-weighted MRI images (iii, iv). ECCL1 H&E shows bipolar cells with long bipolar processes and elongated nuclei (i: 20X, ii: 40X). MRI (iii-coronal): lipoma (arrow) and arachnoid cyst in the posterior fossa; (iv): PA lesion in the hypothalamus (arrow). ECCL2 H&E shows biphasic pattern of PA (i: 20X, ii: 40X). MRI (iii-axial) with thickening of the right sided subcutaneous fat and a lipoma in the cerebellopontine angle (arrows); (iv-axial): lesion located at basal ganglia (arrow), with right side hemispheric atrophy. ECCL3 H&E shows tumor cells radially arranged around the blood vessels with markedly myxoid background; tumor cell nuclei are oval shape with minimal pleomorphism and bipolar cytoplasmic processes, suggesting PMA (i: 200X); a distinct area depicting tumor cells arranged in sheets, showing oval to round monotonous appearance with clear cytoplasm, mimicking oligodendroglia/neurocytes, (also found in PMA) (ii: 200X). MRI (iii-coronal; iv-sagittal) shows an expansive round mass in the third ventricle with enlargement of the lateral ventricle. ECCL4 H&E shows a pseudopalisading necrosis (i: 40x), and abundant microvascular proliferation (ii: 40x). MRI (iii-coronal; iv-axial) showing a ring-enhanced tumor (arrows) extending from the right temporal lobe to basal ganglia. ECCL5 H&E shows typical biphasic ("loose-dense") pattern of PA (i: 20X), and a more "compact" area (ii: 20X). MRI (iii-sagittal; iv-axial) shows a round mass with irregular internal enhancement (arrows) near to the foramen of Monro.

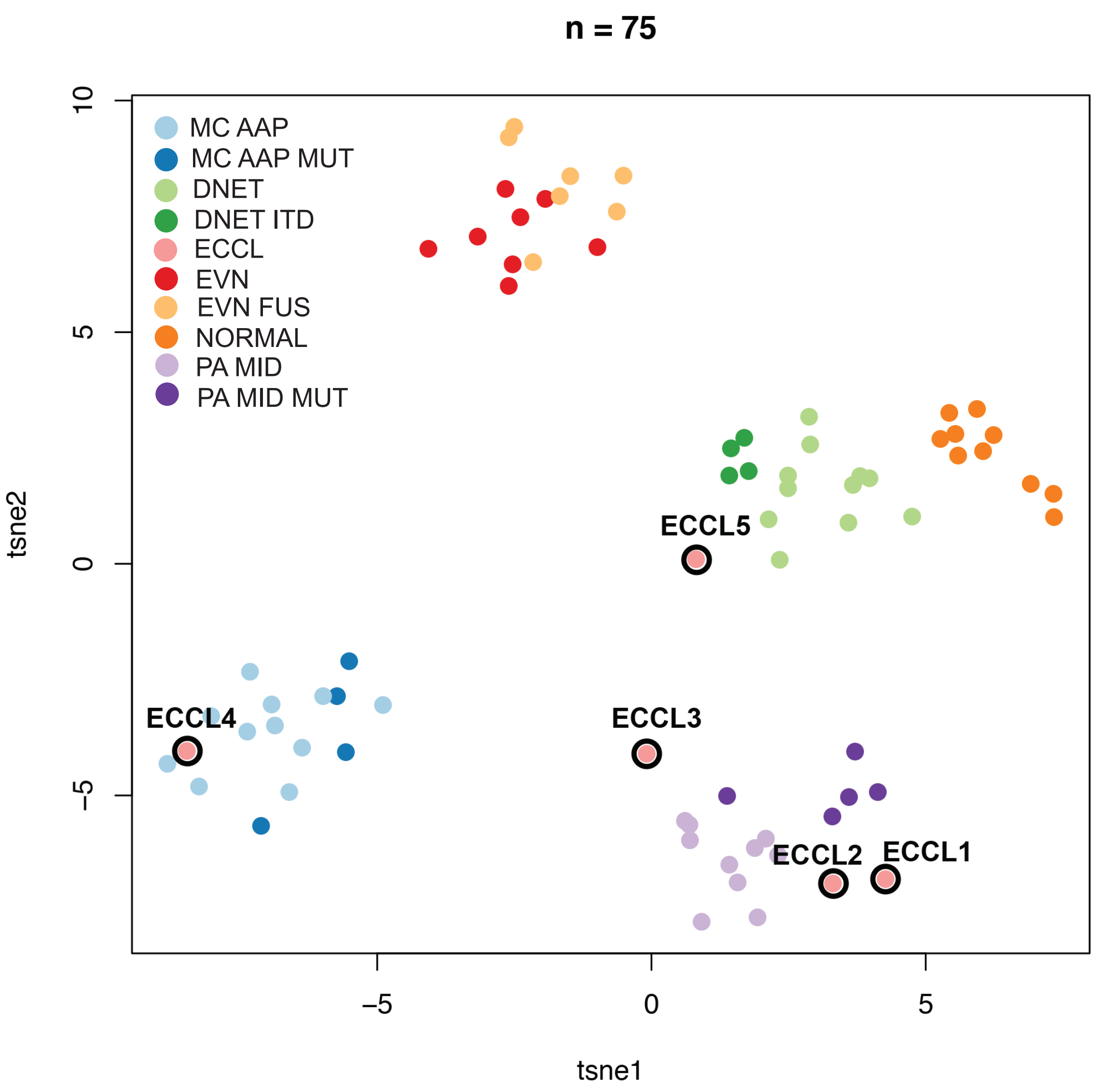

Suppl. Fig. 2 t-SNE analysis of 5 ECCL tumors with 75 references cases from 9 glioma reference classes. Reference classes: MC AAP methylation class anaplastic astrocytoma with piloid features; MC AAP MUT with FGFR1 mutation; DNET dysembryoplastic neuroepithelial tumor; DNET ITD internal duplication of FGFR1; EVN extraventricular neurocytoma; EVN FUS with FGFR1:TACC1 fusion; NORMAL normal brain; PA MID midline pilocytic astrocytoma; PA MID MUT with FGFR1 mutation.

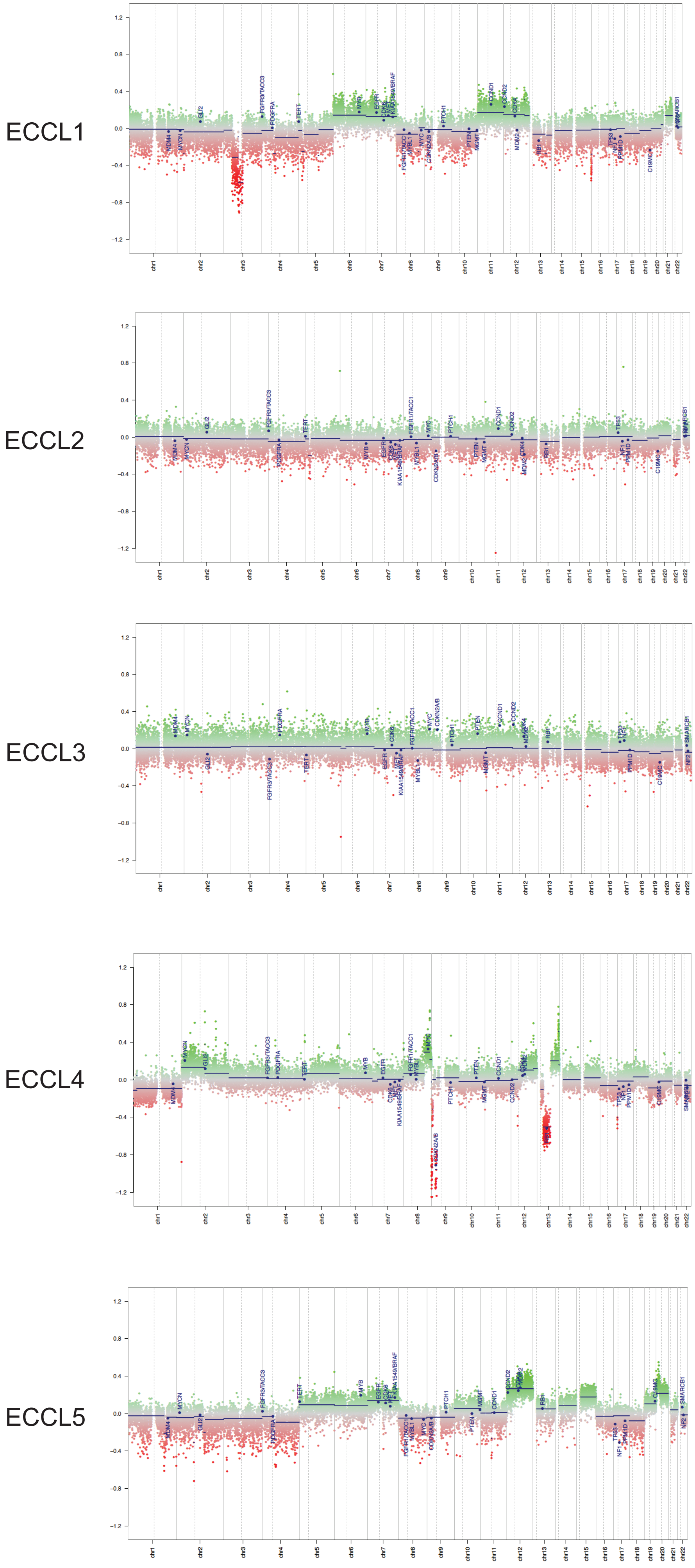

Suppl. Fig 3 Copy number profiles of five ECCL-associated brain tumors

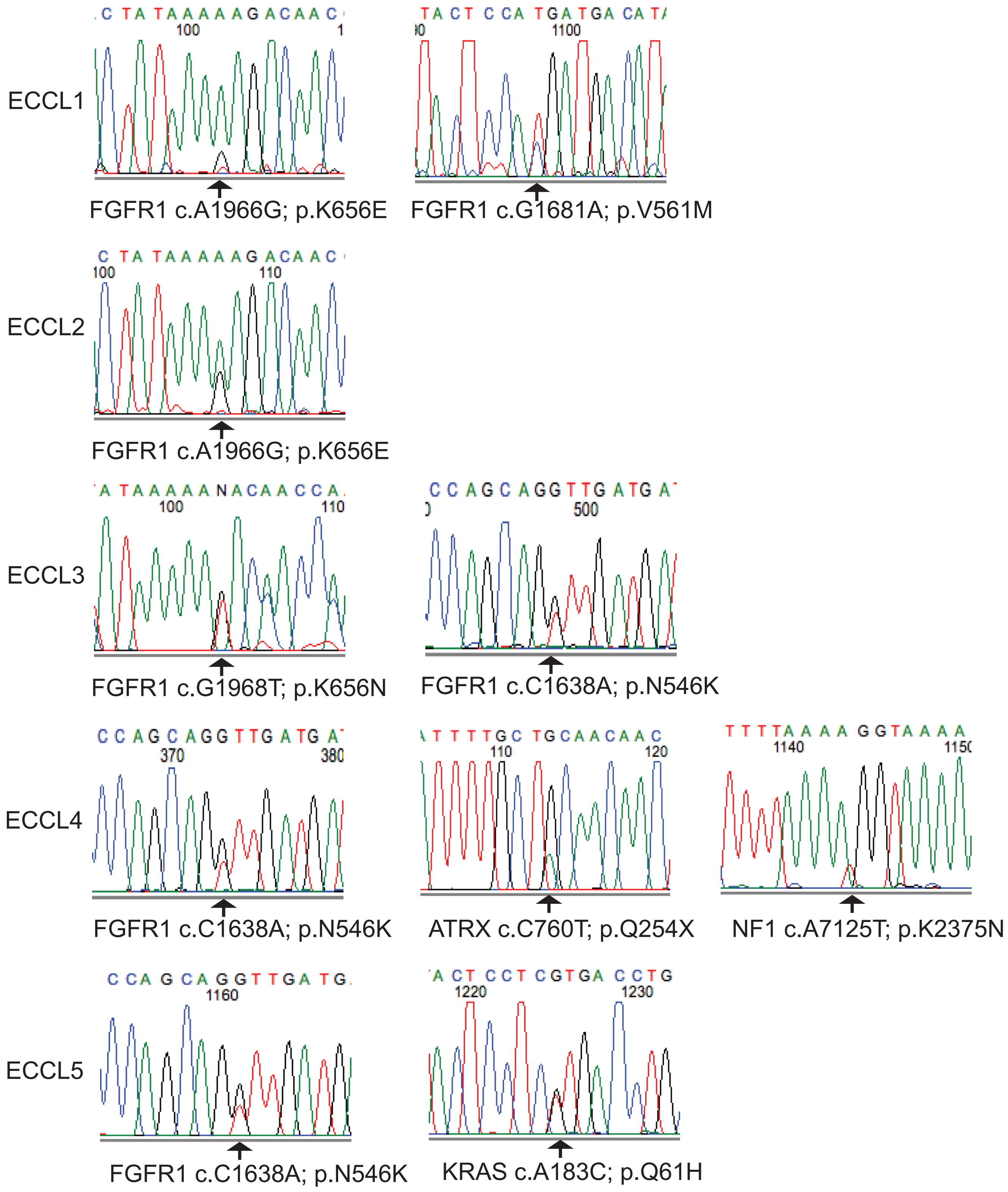

Suppl. Fig 4 Sanger sequencing chromatograms for *FGFR1* mutation validations

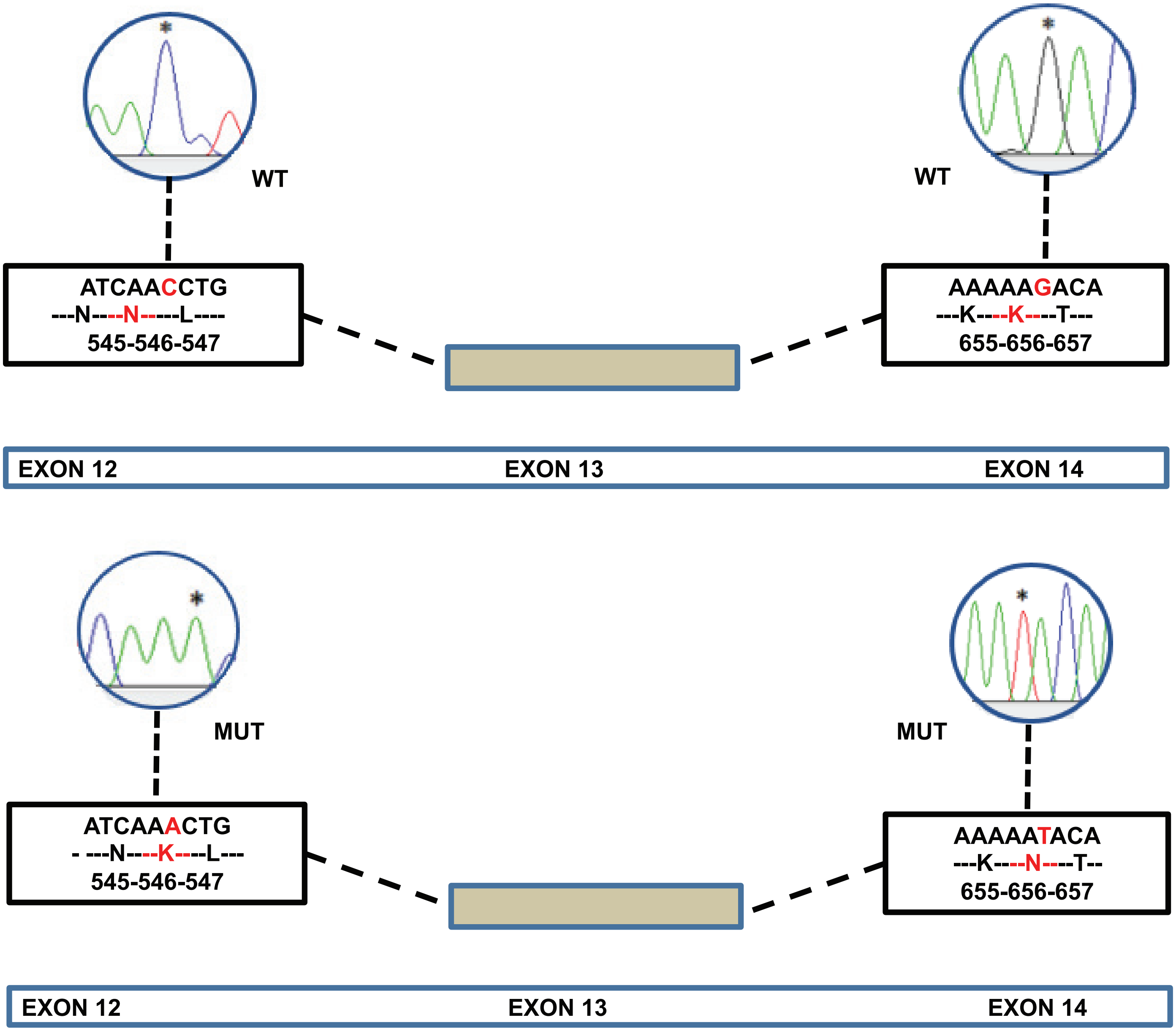

Suppl. Fig 5 Representation of *in-cis FGFR1* N546K and K656N mutations in ECCL3. Eighty five per cent of the colonies showed both mutations *in-cis* with either the two alleles as wild type (WT) or mutant (MUT).

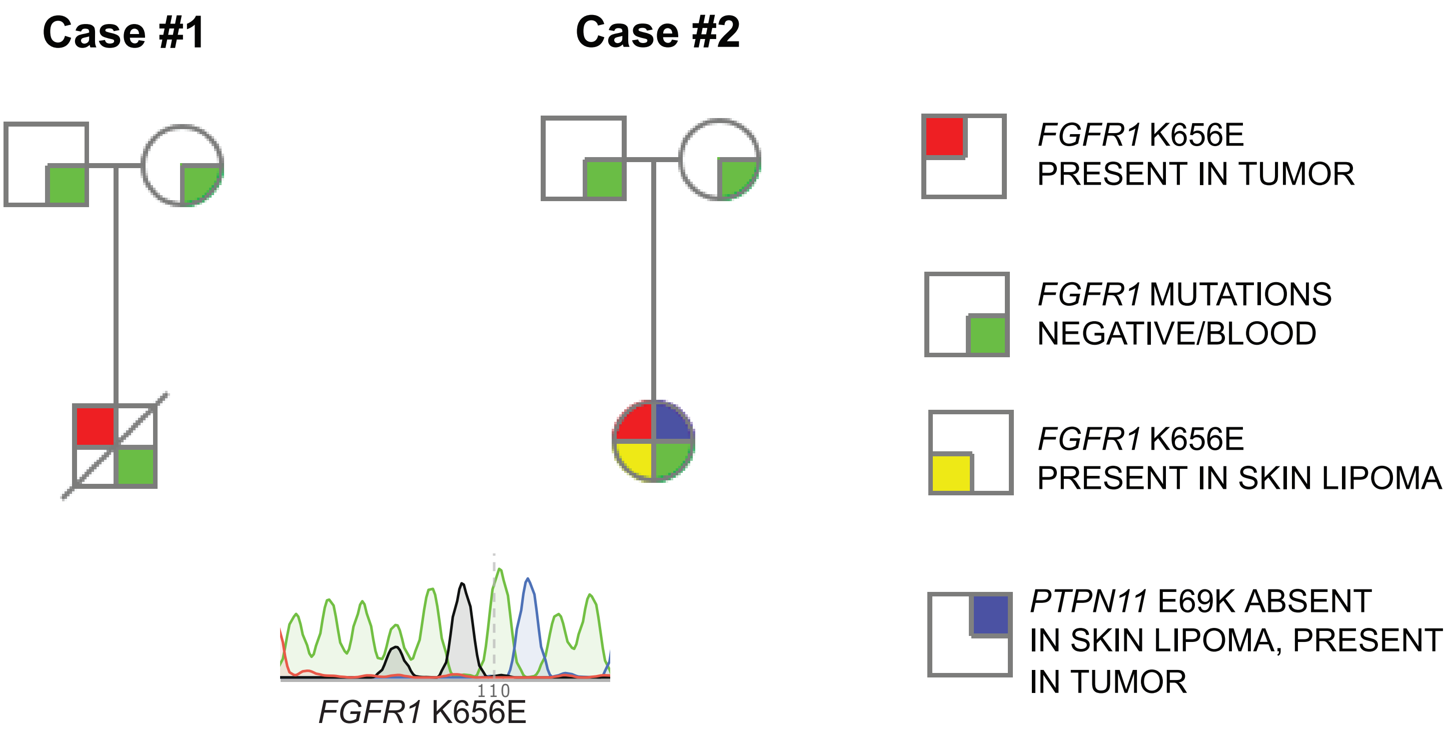

Suppl. Fig 6 Genogram representation of ECCL1 and ECCL2 cases. *FGFR1* N546K and K656E were negative in blood DNA from patients and parents of ECCL1 and ECCL2. ECCL1 and ECCL2 both showed heterozygous *FGFR1* K656E mutation in the tumor DNA. ECCL2 also harbored a somatic *PTPN11* mutation in the tumor. The lipoma harbored the *FGFR1* K656E mutation, but not the *PTPN11* mutation. .
