# Supplementary material for: Methylome analysis and whole-exome sequencing reveal that brain tumors associated with encephalocraniocutaneous lipomatosis are midline pilocytic astrocytomas
Source: Acta Neuropathol. 2018 Aug 24;136(4):657–60. doi: 10.1007/s00401-018-1898-8 (PMC6132939; doi:10.1007/s00401-018-1898-8)
Supplement: Supplementary file 4 — Supplementary material 4 (PDF 179 kb) [file 401_2018_1898_MOESM4_ESM.pdf]

Methylome analysis and whole exome sequencing reveal that brain tumors associated with encephalocraniocutaneous lipomatosis are midline pilocytic astrocytomas

Elvis Terci Valera[1,2*], Melissa K. McConechy[2*], Tenzin Gayden[3*], Barbara Rivera[2], David T. W. Jones[4], Andrea Wittmann[4], HyeRim Han[2], Eric Bareke[5], Hamid Nikbakht[5], Leonie Mikael[3], Rosane Gomes Queiroz[1], Veridiana Kiill Suazo[1], Ji Hoon Phi[6], Seung-Ki Kim[6], Sung-Hye Park[7], Raita Fukaya[8,9], Mi-Sun Yum[10], Tae-Sung Ko[10], Ricardo Santos de Oliveira[11], Helio Rubens Machado[11], María Sol Brassesco[12], Antonio Carlos do Santos[13], Gustavo Novelino Simão[13], Leandra Náira Zambelli Ramalho[14], Luciano Neder[14], Carlos Alberto Scrideli[1], Luiz Gonzaga Tone[#1], Jacek Majewski[#2,5], Nada Jabado[#2,3]

[1]Department of Pediatrics, Ribeirão Preto Medical School, University of São Paulo, Ribeirão Preto, São Paulo, Brazil.

[2]Department of Human Genetics, McGill University, Montreal, Quebec, Canada

[3]Department of Pediatrics, The Research Institute of the McGill University Health Center, Montreal, Quebec, Canada.

[4]Pediatric Glioma Research Group, Hopp Children's Cancer Center at the NCT Heidelberg (KiTZ) and German Cancer Research Center (DKFZ), Heidelberg, 69120, Germany.

[5]McGill University and Genome Quebec Innovation Center, Montreal, Quebec, Canada.

[6]Division of Pediatric Neurosurgery, Seoul National University Children's Hospital, Seoul, Republic of Korea.

[7]Department of Pathology, Seoul National University Children's Hospital, Seoul, Republic of Korea.

[8]Department of Neurosurgery, Shizuoka City Shimizu Hospital, Shizuoka, Japan

[9]Department of Neurosurgery, Fuji Hospital, Aichi, Japan

[10]Division of Pediatric Neurology, Department of Pediatrics, Asan Medical Center Children's Hospital, University of Ulsan College of Medicine, Seoul, Republic of Korea

[11]Division of Pediatric Neurosurgery, Department of Surgery and Anatomy, University Hospital, Ribeirão Preto Medical School, University of São Paulo, Ribeirão Preto, São Paulo, Brazil

[12]Department of Biology, Faculty of Philosophy, Sciences and Letters at Ribeirão Preto, University of São Paulo, Ribeirão Preto, São Paulo, Brazil

[13]Department of Image Science and Medical Physics Center, Internal Medicine, University of São Paulo, Ribeirão Preto, São Paulo, Brazil.

[14]Department of Pathology, Ribeirão Preto Medical School, University of São Paulo, Ribeirão Preto, Brazil.

[*] Equal contribution

[#] co-senior authors

To whom correspondence should be sent:
Elvis Terci Valera, MD, PhD
Department of Pediatrics
Ribeirão Preto Medical School, University of São Paulo, São Paulo, Brazil.

HC Criança - Av. Bandeirantes, 3900, Ribeirão Preto, SP CEP 14048-900 Brazil.

valeraet@gmail.com

Nada Jabado, MD, PhD
Department of Pediatrics
The Research Institute of McGill University Health Center
McGill University
Montreal, QC, H4A 3J1, Canada
nada.jabado@mcgill.ca

Keywords: Encephalocraniocutaneous Lipomatosis; *FGFR1*; RASopathies, genetics, brain tumors; children

Clinical Cohort

The study cohort comprised of five brain tumor specimens, obtained at diagnosis: Fresh-Frozen tissue in 3 cases (ECCL1, ECCL2 and ECCL3) and Formalin-Fixed, Paraffin-Embedded (FFPE) in 2 cases (ECCL4 and ECCL5). Constitutive DNA from peripheral lymphocytes was available for study in three individuals (cases ECCL1, ECCL2 and ECCL4), and peripheral blood DNA from parents in two families (ECCL1 and ECCL2). FFPE samples from skin lipoma and epibulbar choristoma of the eye (ECCL2) were also evaluated. Additional clinical information is available (**Table S1**). All samples were collected after informed consent of patients or their legal guardians.

DNA/RNAextraction

DNA extraction was performed on fresh frozen tissue using the DNeasy Blood & Tissue Kit (Qiagen, Hilden, Germany) following manufacturer's instructions. DNA from FFPE scrolls was extracted using the QIAamp DNA FFPE Tissue Kit (Qiagen®). The truXTRAC FFPE DNA microTUBE Kit (Covaris, Woburn, MA, USA) was used for one ECCL4 due to low DNA quality for WES using different DNA extraction methods. RNA from the PA double mutant (ECCL3) was isolated

using the RNeasy Mini Kit (Qiagen®). Total RNA from FFPE tumor sample from ECCL2 was extracted using RNeasy FFPE (Qiagen®). Reverse transcription (RT) was carried out with Superscript III (Invitrogen, Carlsbad, CA, USA) to synthesize cDNA. All extractions followed manufacture's instructions.

Cloning confirmation of *in cis* mutations for ECCL3

Double-mutant cDNA was amplified for exons 12 to 14 of *FGFR1* (primers and conditions are available upon request). Ligation into a TOPO blunt vector (Invitrogen) was performed according to manufacturer´s recommendations and posterior transformation of OneShot TOP10 *E.coli* competent cells. Bacterial culture was performed overnight in LB agar with kanamycin. A total of 50 colonies were cultured overnight; DNA was purified with QIAamp DNA Mini Kit and followed by Sanger sequencing.

DNA-Methylation array

DNA was extracted from tumors and analyzed for genome-wide DNA methylation patterns using Illumina HumanMethylationEPIC BeadChip arrays. Handling was carried out according to manufacturer's instructions, at the Genomics and Proteomics Core Facility of the German Cancer Research Center (DKFZ). Processing of DNA methylation data was performed with custom approaches as previously described [5,15], and copy number profiles were generated using the 'conumee' package for R (https://www.bioconductor.org/packages/release/bioc/html/conumee.html). Analysis of tumor subgroups was performed using a t-distributed stochastic neighbor embedding (t-SNE)-based approach [17] with a perplexity value of 15. In addition to the two test cases, reference samples of methylation class anaplastic astrocytoma with piloid features (AAP) [12], dysembryoplastic neuroepithelial tumor (DNT), extraventricular neurocytoma (EVN), normal cerebral hemisphere (NORM_HEMI) and midline pilocytic astrocytoma (PA) from the Heidelberg Molecular Neuropathology platform (https://www.molecularneuropathology.org/mnp) were included as a comparison. The suffixes _MUT, _ITD and _FUS indicate samples with *FGFR1* point mutation, internal tandem duplication or fusion (*FGFR1*:*TACC1*) [14], respectively. Hierarchical clustering was performed using the top 3,000 most variable probes (SD), Euclidean distance measures and Ward's clustering method.

Whole exome sequencing

Whole exome sequencing (WES) was performed on 200ng of DNA from all five tumor cases, and on three cases of peripheral blood DNA from matched-patients using the Agilent SureSelect exome capture kit v.5 (Agilent Technologies, Santa Clara, CA, USA); the libraries were sequenced on an Illumina HiSeq 2500 (Illumina, San Diego, CA, USA) with paired-end 125bp reads at the McGill University and Genome Quebec Innovation Center. Sequences were aligned to the human reference genome (UCSC hg19 assembly) using the BWA (Burrows-Wheeler Aligner v. 0.7.7) algorithm [9]. Duplicate reads were removed using Picard tools v.2.1.0. The Genome Analysis Toolkit (GATK v.3.7) RealignerTargetCreator/IndelRealigner tools were used to remove artifacts near Indels (small insertions or deletions) known to occur in the initial alignment process [3,11]. Following re-alignment, base quality scores were recalibrated using GATK BaseRecalibrator tool and variant calling was performed using a combination of GATK Haplotype caller and SAMtools version 1.3 [10] for SNP calling and GATK Haplotype caller alone for Indel calling. For each called position, a minimum of two variant reads and >20% SNV (Single-nucleotide variants) or >15% Indels variant reads were considered. Variants were annotated using ANNOVAR for functional annotations [18] and custom scripts to add predicted conservation scores (GERP[2] and PhastCons tools[13]), pathogenicity scores (SIFT[7], PolyPhen2[1] and Combined Annotation Dependent Depletion [CADD v.1.3][6]), and population allele frequencies (in-house wide[cancer/non-cancer] control database of 2734 exomes and rare-disease control database of 1572 exomes, EVS[16], ExAC v.0.3[8]), to determine the effect of called variants on genes, transcripts, and protein sequence. To prioritize the annotated variants, we applied a filtering strategy to look for coding, non-synonymous variants, by retaining those with 1000 Genomes [4], ExAC with a Minor Allelic Frequency (MAF) of less than 0.005%.

PCR, Sanger and targeted amplicon sequencing

Sanger sequencing for screening the two *FGFR1* mutations for ECCL diagnosis (p.N546K and p.K656E) were performed on PCR products from tumor DNA samples (n=5), peripheral blood DNA from affected individuals (n=3), blood DNA from two family clusters (n=4) and on two different tissues available for

ECCL2. Additional primers (*FGFR1* p.V561M, *FGFR1* p.K656N, *KRAS* p.Q61H, *NF1* p.K2375N, and *ATRX* p.Q254X) were designed to validate FGFR1/RAS/MAPK mutations and additional pathogenic mutations of interest found by WES analysis. M13 tailed primers were designed using Primer3 software (http://bioinfo.ut.ee/primer3-0.4.0/). Primer sequences and PCR conditions are available upon request. PCR products were submitted to bidirectionally sequencing on an ABI 3730XL DNA Analyzer (Applied Biosystems, USA). The resulting chromatograms were visualized by SnapGene software (version 4.1).

Targeted amplicon sequencing was performed on PCR products amplified with gene-specific CS1/CS2-tagged primers (CS1 5'ACACTGACGACATGGTTCTACA3', CS2 5'TACGGTAGCAGAGACTTGGTCT3'). PCR products were barcoded and pooled for sequencing on the Illumina MiSeq (Illumina, San Diego, CA, USA) with paired-end 250bp reads at the McGill University and Genome Quebec Innovation Center. Resulting bam files with read counts were obtained from the Nanuq platform.

AUTHOR CONTRIBUTIONS

E.T.V, M.K.M., T.G., B.R., D.T.W.J, A.W, H.H., performed experiments. E.B., H.N., J.M., performed bioinformatic analyses. E.T.V., T.G., M.K.M., B.R., D.T.W.J, A.W., H.H., L.M., performed data analyses and generated the text and figures. L.M., R.G.Q., V.K.S., J.H.P., S.K., R.F., M.Y., S.H.P., T.K., R.S.O., H.R.M., M.S.B., A.C.S., G.N.S., L.N.Z.R., L.N., C.A.S., L.G.T. collected data and provided patient materials. E.T.V, M.K.M, T.G., drafted the manuscript. J.M., L.G.T. and N.J. provided leadership for the project. All authors contributed to the final manuscript.

References

1 Adzhubei IA, Schmidt S, Peshkin L, Ramensky VE, Gerasimova A, Bork P, et al (2010) A method and server for predicting damaging missense mutations. Nature methods 7: 248-249 Doi 10.1038/nmeth0410-248

2 Cooper GM, Stone EA, Asimenos G, Program NCS, Green ED, Batzoglou S, et al (2005) Distribution and intensity of constraint in mammalian genomic sequence. Genome Res 15: 901-913 Doi 10.1101/gr.3577405

3 DePristo MA, Banks E, Poplin R, Garimella KV, Maguire JR, Hartl C, et al (2011) A framework for variation discovery and genotyping using next-generation DNA sequencing data. Nat Genet 43: 491-498 Doi 10.1038/ng.806

4 Genomes Project C, Abecasis GR, Auton A, Brooks LD, DePristo MA, Durbin RM, et al (2012) An integrated map of genetic variation from 1,092 human genomes. Nature 491: 56-65 Doi 10.1038/nature11632
5 Hovestadt V, Remke M, Kool M, Pietsch T, Northcott PA, Fischer R, et al (2013) Robust molecular subgrouping and copy-number profiling of medulloblastoma from small amounts of archival tumour material using high-density DNA methylation arrays. Acta neuropathologica 125: 913-916 Doi 10.1007/s00401-013-1126-5
6 Kircher M, Witten DM, Jain P, O'Roak BJ, Cooper GM, Shendure J (2014) A general framework for estimating the relative pathogenicity of human genetic variants. Nat Genet 46: 310-315 Doi 10.1038/ng.2892
7 Kumar P, Henikoff S, Ng PC (2009) Predicting the effects of coding non-synonymous variants on protein function using the SIFT algorithm. Nat Protoc 4: 1073-1081 Doi 10.1038/nprot.2009.86
8 Lek M, Karczewski KJ, Minikel EV, Samocha KE, Banks E, Fennell T, et al (2016) Analysis of protein-coding genetic variation in 60,706 humans. Nature 536: 285-291 Doi 10.1038/nature19057
9 Li H, Durbin R (2009) Fast and accurate short read alignment with Burrows-Wheeler transform. Bioinformatics 25: 1754-1760 Doi 10.1093/bioinformatics/btp324
10 Li H, Handsaker B, Wysoker A, Fennell T, Ruan J, Homer N, et al (2009) The Sequence Alignment/Map format and SAMtools. Bioinformatics 25: 2078-2079 Doi 10.1093/bioinformatics/btp352
11 McKenna A, Hanna M, Banks E, Sivachenko A, Cibulskis K, Kernytsky A, et al (2010) The Genome Analysis Toolkit: a MapReduce framework for analyzing next-generation DNA sequencing data. Genome Res 20: 1297-1303 Doi 10.1101/gr.107524.110
12 Reinhardt A, Stichel D, Schrimpf D, Sahm F, Korshunov A, Reuss DE, et al (2018) Anaplastic astrocytoma with piloid features, a novel molecular class of IDH wildtype glioma with recurrent MAPK pathway, CDKN2A/B and ATRX alterations. Acta neuropathologica 10.1007/s00401-018-1837-8: Doi 10.1007/s00401-018-1837-8
13 Siepel A, Bejerano G, Pedersen JS, Hinrichs AS, Hou M, Rosenbloom K, et al (2005) Evolutionarily conserved elements in vertebrate, insect, worm, and yeast genomes. Genome Res 15: 1034-1050 Doi 10.1101/gr.3715005
14 Sievers P, Stichel D, Schrimpf D, Sahm F, Koelsche C, Reuss DE, et al (2018) FGFR1:TACC1 fusion is a frequent event in molecularly defined extraventricular neurocytoma. Acta neuropathologica 136: 293-302 Doi 10.1007/s00401-018-1882-3
15 Sturm D, Witt H, Hovestadt V, Khuong-Quang D-A, Jones David TW, Konermann C, et al (2012) Hotspot Mutations in H3F3A and IDH1 Define Distinct Epigenetic and Biological Subgroups of Glioblastoma. Cancer Cell 22: 425-437 Doi 10.1016/j.ccr.2012.08.024
16 Tennessen JA, Bigham AW, O'Connor TD, Fu W, Kenny EE, Gravel S, et al (2012) Evolution and functional impact of rare coding variation from deep sequencing of human exomes. Science 337: 64-69 Doi 10.1126/science.1219240
17 van der Maarten L, Hinton G (2008) Visualizing High-Dimensional Data Using t-SNE. Journal of Machine Learning Research 9: 2579-2605

18 Wang K, Li M, Hakonarson H (2010) ANNOVAR: functional annotation of genetic variants from high-throughput sequencing data. Nucleic Acids Res 38: e164 Doi 10.1093/nar/gkq603
